# Development of best evidence-based practice protocols for central venous catheter placement and maintenance to reduce CLABSI

**DOI:** 10.1097/MD.0000000000038652

**Published:** 2024-07-05

**Authors:** Xiu-wen Chi, Ru He, Xiao-heng Wu, Li-juan Wu, Yuan-li Yang, Zhen Huang

**Affiliations:** a School of Nursing, Guangdong Medical University, Dongguan, China; b Nursing Department, Longgang Central District Hospital, Shenzhen, China; c Department of Intensive Care Unit, Longgang Central District Hospital, Shenzhen, China; d Office of Shenzhen Clinical College, Guangzhou University of Chinese Medicine, Longgang Central District Hospital, Shenzhen, China.

**Keywords:** central line-associated blood stream infection, central venous catheter, intensive care unit, nurses, PARIHS, placement

## Abstract

Although evidence-based interventions can reduce the incidence of central line-associated bloodstream infection (CLABSI), there is a large gap between evidence-based interventions and the actual practice of central venous catheter (CVC) care. Evidence-based interventions are needed to reduce the incidence of CLABSI in intensive care units (ICU) in China. Professional association, guidelines, and database websites were searched for data relevant to CLABSI in the adult ICUs from inception to February 2020. Checklists were developed for both CVC placement and maintenance. Based on the Integrated Promoting Action on Research Implementation in Health Services framework, a questionnaire collected the cognition and practice of ICU nursing and medical staff on the CLABSI evidence-based prevention guidelines. From January 2018 to December 2021, ICU CLABSI rates were collected monthly. Ten clinical guidelines were included after the screening and evaluation process and used to develop the best evidence-based protocols for CVC placement and maintenance. The CLABSI rates in 2018, 2019, and 2020 were 2.98‰ (9/3021), 1.83‰ (6/3276), and 1.69‰ (4/2364), respectively. Notably, the CLABSI rate in 2021 was 0.38‰ (1/2607). In other words, the ICU CLABSI rate decreased from 1.69‰ to 0.38‰ after implementation of the new protocols. Additionally, our data suggested that the use of ultrasound-guidance for catheter insertion, chlorhexidine body wash, and the use of a checklist for CVC placement and maintenance were important measures for reducing the CLABSI rate. The evidence-based processes developed for CVC placement and maintenance were effective at reducing the CLABSI rate in the ICU.

## 1. Introduction

A central line-associated bloodstream infection (CLABSI) is a primary bloodstream infection that develops in a patient with a central line in place without evidence of an infectious source at any other place.^[1,2]^ CLABSI occurs when these 3 criteria exist: Clinical signs of infection, for example, fever, rigors, altered mental status, hypotension; no alternate source of bloodstream infection; positive blood culture from a peripheral vein with any one of the following: catheter tip/segment culture that matches organism grown from blood; at least threefold higher number of organisms grown from the catheter versus the peripheral blood culture on simultaneously drawn cultures; and growth from the catheter-drawn blood culture occurs at least 2 hours before growth of the same organism from a percutaneously-drawn blood culture.^[3]^ CLABSI often occurs in patients being treated in an intensive care units (ICU), and is a leading cause of death in ICU patients.^[4,5]^ CLABSI results in the death of thousands of people each year, and increases healthcare costs by billions of dollars.^[6]^ How to effectively prevent CLABSIs has always been an important topic of medical research.

Several studies have demonstrated that the incidence of CLABSIs can be reduced through evidence-based interventions. The Keystone ICU Evidence-based Intervention Program initiated by the Michigan Health and Hospital Association reduced the median incidence of CLABSIs in 103 ICUs from 2.7 infections/1000 catheter days at baseline to zero at 3 months after beginning the program.^[7]^ Jamous et al^[8]^ demonstrated that the incidence of CLABSI decreased from 17 cases per thousand to zero over 10 months after a quality improvement program was initiated. Doctor Pearlman has written that quality improvement methods can effectively reduce the incidence of CLABSIs, and that it is possible to achieve a zero CLABSI rate.^[9]^

Although evidence-based interventions can reduce the incidence of CLABSIs, there is a large gap between evidence-based interventions and the actual practice of central venous catheter (CVC) care in the ICU. Ullman et al^[10]^ reported a large gap between evidence-based practices and nursing knowledge of CVC care in pediatric ICUs.^[11]^ Labeau et al^[12]^ found that European ICU nurses have opportunities to optimize their knowledge of CVC-related infection prevention.

Xu et al^[13]^ reported that the mean catheter flow rate in the ICUs of 25 hospitals in Hubei Province, China, was 1.23‰ in 2016, and the incidence of CLABSI in 10 hospitals, including 3 university-affiliated hospitals, in Guangdong Province, China, was about 3‰ in 2018. Therefore, evidence-based interventions are needed to reduce the incidence of CLABSI in ICUs in China.

The Promoting Action on Research Implementation in Health Services (PARIHS) framework was developed 2 decades ago. The framework has been widely used, tested, reviewed, and refined. The Integrated PARIHS (i-PARIHS) framework facilitates positioning as a positive factor for implementation, and assesses and adjusts innovations to be implemented with intended recipients in local, organizational, and broader system contexts. Facilitation is implemented through a network of novice, experienced, and expert facilitators, who apply a range of support skills and improvement strategies to build the implementation process, engage and manage relationships among key stakeholders, and identify and negotiate implementation obstacles in context. Researchers wanted to act as facilitators to promote the implementation of clinical evidence.

Therefore, the aim of the current study was to construct a best evidence-based practice plan for CVC maintenance, and evaluate the effect of its application through the pilot program in an ICU. Our study will apply the i-PARIHS theoretical framework to promote evidence-based nursing practice and assess the incidence of CLABSI in the ICU.

## 2. Methods

**Figure 1.**
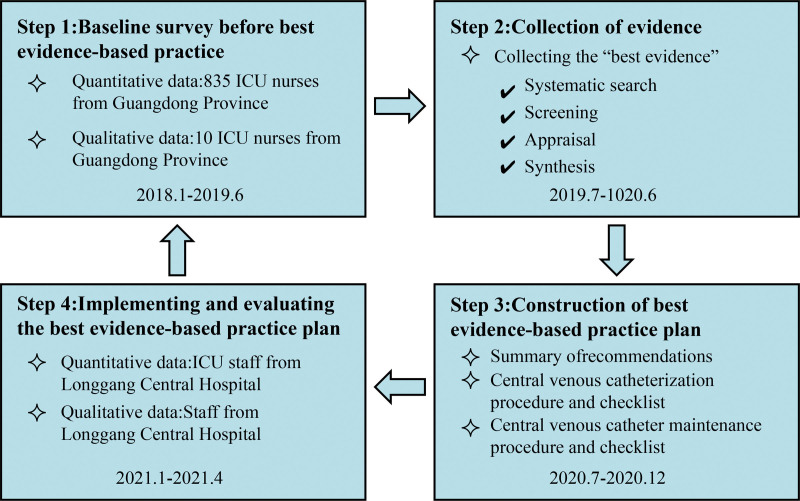
Flowchart of the construction and implementation of best evidence-based practice plan. ICU = intensive care unit.

This was an interventional hybrid study, and the research process is shown in Figure 1. Data for the study were collected by interviewing the adult ICU staff of Longgang Central Hospital, Shenzhen, Guangdong, China. The questionnaire used collected quantitative data on the knowledge and practice of CLABSI evidence-based prevention guidelines of the ICU nurses, and was administered at 3 time points: before the project, before the application of evidence, and after the application of evidence. This study was approved by the Medical Ethics Committee of Longgang Central District Hospital, Shenzhen.

The Planning for Implementation: A Facilitation Checklist, part of the i-PARIHS framework, was used as an interview outline to evaluate the implementation of the program.^[14]^

### 2.1. Baseline survey before implementation of the evidence-based plan

#### 2.1.1. Quantitative data

Investigation of the knowledge and practice of ICU nurses of CLABSI evidence-based prevention guidelines disclosed the knowledge and practice of CLABSI evidence-based prevention guidelines. The mean score of 11 questions related to evidence-based guidelines for preventing CLABSIs was 4.02.^[15]^ This finding was consistent with the recommendation of National Health authorities that hospitals should adopt policies to train ICU nurses in the prevention of CLABSIs.

#### 2.1.2. Qualitative data

Evidence regarding CVC maintenance was obtained via a literature search, a structured interview outline was created based on the evidence collected, and 10 ICU nurses from different hospitals in Guangdong Province, China, were interviewed. The incidence of CLABSI in all visited ICUs was around 3‰. Anti-infective catheters were used in 3 ICUs. In 2 ICUs, the sterile cavity towel during CVC insertion was smaller. Two ICUs changed polyurethane transparent dressings every 3 days. Five ICUs did not strictly require nurses to wear gloves when changing dressings. Five ICUs used heparin water to seal catheters. Three ICUs used iodine povidone as an antiseptic solution for tube placement and dressing change. Drug delivery devices and pressure monitoring devices were not changed at the same time in different ICUs. Two ICUs routinely changed CVCs. The results confirmed those of other reports that there is a gap between practice and current evidence for CVC maintenance in the ICU, and that evidence-based nursing measures should be adopted to reduce the incidence of CLABSI and improve patient outcomes.^[16]^

### 2.2. Collection of evidence

#### 2.2.1. Systematic literature search

Professional association websites, guidelines websites, and databases were searched from inception to February 2020 for evidence-based information related to the prevention of CLABSIs. Keywords used included: critical care, intensive care unit, ICU, intensive care, central venous catheter, catheterization, central venous, central venous catheter, CVC, catheter-related infections, infection, central line, critical illness, critical patient care, critical care outcomes, and central venous catheter infection.

The inclusion criteria for evidence-based information/guidelines for the prevention of CLABSI were: Chinese or English language; the scope of the information was related to CLABSI; the information provided guidance for interventions to reduce the rate of CLABSIs; and the population to which the guidelines addressed was adult. The exclusion criteria were: repeated information; and information that interpreted guidelines and aftereffect evaluation of guidelines.

#### 2.2.2. Screening

Screening of guidelines/information consisted of 2 steps. In the first step, the title of the article and abstract was reviewed by a nursing teacher and a chief physician to eliminate irrelevant topics, duplications, object inconsistencies, and language inconsistencies, as well as articles in which the full text was unavailable. The number of articles excluded and the reasons for exclusion were recorded. In step 2, the full text of the articles that met the inclusion criteria were reviewed. Any articles that did not meet the inclusion criteria were excluded, and the remaining articles/guidelines were included for further evaluation.

#### 2.2.3. Appraisal

The Appraisal of Guidelines for Research and Evaluation (AGREE) Instrument was used to evaluate guidelines independently by 2 evaluators. Each item was scored on a scale of 1 to 7: 7 points that were given if there was full compliance with the requirements of the item, and 1 point was given for complete noncompliance or information not mentioned in the guidelines. After the evaluation of the guidelines, a preliminary collation was performed and items which varied greatly in score were discussed to ensure that there was no misevaluation due to omission of information. Recommendations for use of a guideline were based on the standardized percentage of 6 fields^[17]^: standardized percentage of 6 fields > 50%, strongly recommended; standardized percentage of 4 or more fields > 50%, recommend; standardized percentage of 3 or more fields >50%, not recommended or uncertain; standardized percentage >50% in 2 or fewer fields, not recommend.

#### 2.2.4. Synthesis

Content analysis of the included clinical practice guidelines was performed. Through content analysis, a comprehensive table of evidence and recommendations was compiled, which included the primary evidence on the implementation and management of CVC maintenance in the ICU. Units of analysis or focus issues, based on the topic and developed criteria for categorization, were categorized. For each guideline, the most recent recommendation or evidence-based recommendation was given priority. Any conflicts between the recommendations of different guidelines were extracted separately and reviewed by experts.

### 2.3. Construction of the evidence-based practice plan

#### 2.3.1. Summary of recommendations

Based on the summaries of 10 guidelines that were reviewed and re-reviewed by clinical experts from Longgang Central Hospital, a summary of recommendations of evidence-based practice for central venous catheter maintenance in the ICU was developed. The quality of evidence in the summaries was graded using the system developed by the United States Preventive Services Task Force.^[18]^ The final level of evidence grading was based on the latest version of the Grading of Recommendations Assessment, Development and Evaluation (GRADE) system proposed by the GRADE Working Group in 2011. The GRADE system simplifies levels of recommendation “strong recommendation” and “weak recommendation,” and adds “not recommended” to screen for recommendation items that are temporarily inconsistent with the clinical situation.^[19]^

#### 2.3.2. Procedures and checklists

From July 2019 to December 2020, an evidence-based practice plan was developed, which included evidence summary, video interpretation of evidence summary, procedures and checklists for catheter indwelling and catheter maintenance, etc. From December 21, 2020 to December 31, 2021, the evidence-based practice program was distributed to ICU medical staff for learning. In January 2021, the checklists were applied for pre-intervention and adjusted based on clinical feedback. From February to April 2021, the checklists were applied for formal intervention. The checklists were filled out by the nurse on duty. The CVC maintenance process developed by Tao et al^[20]^ was used as the basis, and additional evidence-based processes were added to it. In addition, a checklist was developed for the central venous catheter implantation procedure.

### 2.4. Implementing and evaluating the evidence-based practice plan

#### 2.4.1. Quantitative data

As indicated previously, ICU nurses at Longgang Central Hospital were questioned about their knowledge of evidence-based CLABSI prevention guidelines before and after application of the newly developed evidence-based guidelines. ICU CLABSI infection rates were collected monthly from January 2018 to December 2021.

#### 2.4.2. Qualitative data

The “Planning for Implementation: A Facilitation Checklist” was used to conduct interviews with the ICU medical staff of Longgang Central Hospital in order to evaluate the implementation of the innovation.

## 3. Results

### 3.1. Collection of evidence

**Figure 2.**
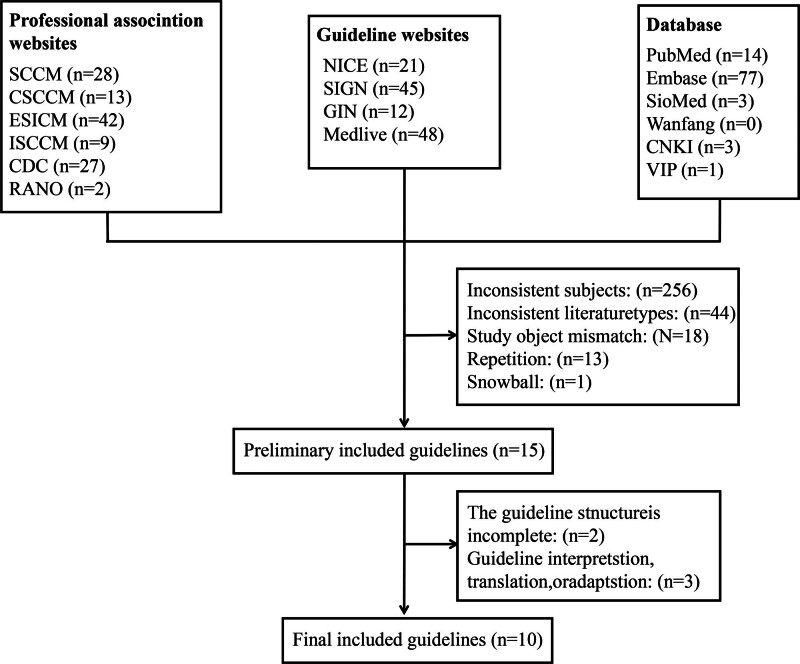
Flow diagram of guideline screening and inclusion. CDC = Centers for Disease Control and Prevention, CNKI = XXXXX, CSCCM = Chinese Society of Critical Care Medicine, ESICM = European Society of Intensive Care Medicine, GIN = Guidelines International Network, ISCCM = Indian Society of Intensive Care Medicine, RNAO = Registered Nurses’ Association of Ontario, SCCM = Society of Critical Care Medicine, SIGN = Scottish Intercollegiate Guidelines Network, VIP = XXXX.

After the aforementioned screening and evaluation process, 10 evidence-based guidelines were ultimately included in the study. The latest published versions ranged from publication between 2001 and 2021, and the guidelines were from the United States, Canada, China, the Asia-Pacific region, Sweden, the United Kingdom, and Italy.^[5,17,21–28]^ The screening and collection process is shown in Figure 2, and the names and publication years of the included guidelines are shown in Table 1. AGREE II scores of the included guidelines in the 6 areas are summarized in Table 2. In 1 guideline published in Chinese, the AGREE II score in 2 areas had a standardized percentage of >50%.

**Table 1:** Summary of the guidelines used to develop the evidence-based practice plan.

Number	Guideline name	Year published	Year updated year	Publisher/author
G1	Guidelines for the Prevention of Intravascular Catheter-Related Infections, 2011	2002	2017	SCCM
G2	Care and Maintenance to Reduce Vascular Access Complications	2005	2021	RANO
G3	Strategies to Prevent Central Line–Associated Bloodstream Infections in Acute Care Hospitals, 2014 Update	2008	2014	SHEA
G4	Guidelines for the prevention and treatment of intravascular catheter-related infections (2007)	2007	Not updated	CSCCM
G5	Practice Guidelines for Central Venous Access 2020	2012	2020	ASA
G6	APSIC guide for prevention of Central Line Associated Bloodstream Infections (CLABSI)	2016	Not updated	ASPIC
G7	Clinical Guidelines on Central Venous Catheterization	2014	Not updated	SFAI
G8	National Evidence-Based Guidelines for Preventing Healthcare-Associated Infections in NHS Hospitals in England	2001	2014	NHS
G9	3M Tegaderm CHG IV securement dressing for central venous and arterial catheter insertion sites	2015	Not updated	NICE
G10	International Evidence-Based Recommendations on Ultrasound-Guided Vascular Access	2012	Not updated	ILC-USVA

ASA = American Society of Anesthesiologists, ASPIC = Asia-Pacific Society for infection Control, CSCCM = Chinese Society of Critical Care Medicine, ILC-USVA = International Liaison Committee on Ultrasound Vascular Access, NHS = National Health Service, NICE = National Institute for Health and Care Excellence, RNAO = Registered Nurses’ Association of Ontario, SCCM = Society of Critical Care Medicine, SFAI = Swedish Society of Anaesthesiology and Intensive Care Medicine, SHEA = Society for Healthcare Epidemiology of America.

**Table 2:** Evaluation of guidelines in 6 areas using the Appraisal of Guidelines for Research and Evaluation tool.

Clinical guideline	Domain						Number of fields ≥ 50%
	Scope and purpose (%)	Participants (%)	Rigorism (%)	Clarity (%)	Applicability (%)	Editorial independence (%)	
G1	86.11	66.67	54.17	100.00	68.75	50.00	6
G2	100.00	66.67	94.79	100.00	87.50	45.83	5
G3	100.00	44.44	36.46	100.00	93.75	50.00	4
G4	66.67	19.44	50.00	100.00	25.00	0.00	3
G5	100.00	66.67	85.42	100.00	45.83	50.00	5
G6	66.67	16.67	57.29	100.00	100.00	100.00	4
G7	100.00	19.44	75.00	100.00	25.00	50.00	4
G8	100.00	52.78	100.00	100.00	43.75	79.17	5
G9	100.00	80.56	84.38	66.67	75.00	12.50	5
G10	100.00	91.67	50.00	100.00	58.33	0.00	5

Clinical guideline numbers refer to those in Table 1.

### 3.2. Construction of the evidence-based practice plan

A checklist for the central venous catheter implantation procedure is shown in Table S1, Supplemental Digital Content, http://links.lww.com/MD/N63, and a checklist for central line maintenance is shown in Table S2, Supplemental Digital Content, http://links.lww.com/MD/N64. Maintenance management processes for CVCs have been previously reported.^[20]^ The checklist of central venous catheter placement is completed in 2 copies. The checklist for central venous catheter maintenance completed 166 copies.

### 3.3. Quantitative data

Responses to the “Knowledge of CLABSI Prevention Questionnaire” are shown in Table 3. With the exception of the 2 knowledge points (Question 6 and Question 7), the percentage of correct knowledge point responses increased. The results of the “Behavior Questionnaire on Prevention of CLABSIs” are shown in Table 4; All but 2 behaviors (Behaviour 3 and Question 8) were upgraded.

**Table 3:** Responses to the “Knowledge of CLABSI Prevention Questionnaire.”

Question number	Question	Percent correct (n = 21) 2019/July (%)	Percent correct (n = 45) 2020/November (%)	Percent correct (n = 35) 2021/July (%)
1	It is recommended to replace CVCs routinely.	28.57	57.78	45.71
2	In settings with a high rate of catheter-related infections it is recommended to use a CVC coated or impregnated with an antiseptic agent.	14.29	51.11	68.57
3	It is recommended to change the dressing on the catheter insertion site…	57.14	73.33	65.71
4	It is recommended to cover the catheter insertion site with…	19.05	26.67	31.43
5	It is recommended to apply an antibiotic ointment at the insertion site of the CVC.	28.57	53.33	40
6	When blood, blood products, or lipid emulsions are administered through a CVC, it is recommended to replace the administration set…	85.71	88.89	71.43
7	When liquids other than blood, blood products, or fat emulsions are administered continuously the administration set should be replaced.	14.29	11.11	8.57
8	It is recommended to use an antiseptic agent to clean the access hub or connector before the connection of the administration set, or after unscrewing the dead-end cap that closes the catheter.	42.86	71.11	62.86
9	When manipulating the catheter insertion site and hubs, it is recommended…	80.95	91.11	82.86
10	It is recommended to replace pressure transducers and tubing routinely...	33.33	51.11	51.43

CVC = central venous catheter.

**Table 4:** Results of the “Behavior Questionnaire on Prevention of CLABSI.”

No.	How frequently are these practices used in your facility?	Percent correct (n = 21) 2019/July (%)	Percent correct (n = 45) 2020/November (%)	Percent correct (n = 35) 2021/July (%)
1	Maximum barrier precautions (cap, mask, sterile gown, sterile gloves, and a sterile full body drape)	90.48	95.56	88.57
2	2 chlorhexidine gluconate for antisepsis of the insertion site	71.43	88.89	85.71
3	Use of suture-less securement devices	42.86	37.78	42.86
4	Use of sterile, transparent, semi-permeable dressing to cover the catheter site	71.43	100.00	85.71
5	Transparent dressing replaced at least every 7 days	80.95	88.89	91.43
6	Administration sets replaced no more frequently than at 96-hour intervals, but at least every 7 days	71.43	75.56	74.29
7	Prompt removal of catheter when no longer essential	80.95	93.33	82.86
8	Routine catheter changes even if there is no suspicion of a CLABSI	38.10	26.67	37.14

Percent correct questions 1–7 = (n [mostly] + n [always])/N.

Percent correct question 8 = (n [never] + n [rarely])/N.

CLABSI = central line-associated bloodstream infection.

### 3.4. CLABSI rates

**Figure 3.**
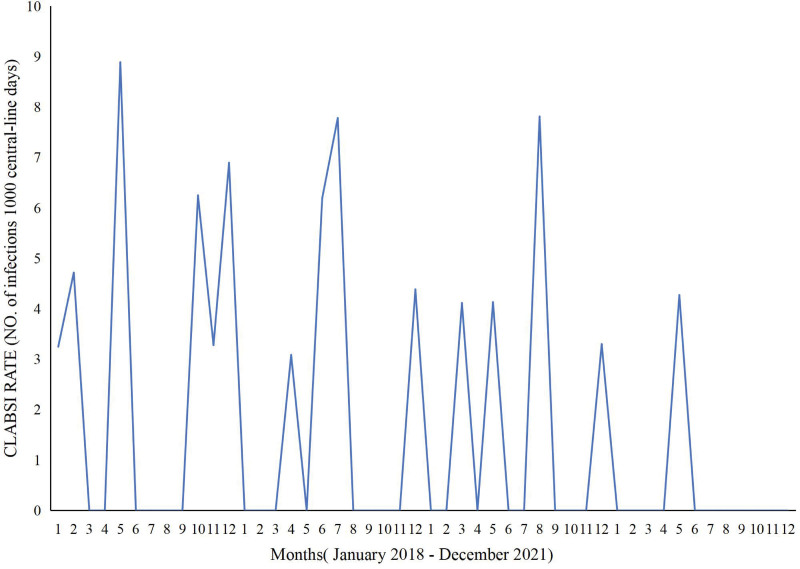
CLABSI rates in the intensive care unit of Longgang Central Hospital from January 2018 to December 2021. CLABSI = central line-associated bloodstream infection.

Monthly CLABSI infection rates in the ICU of Longgang Central Hospital from January 2018 to December 2021 are shown in Figure 3. The Central-line days (Number of CLABSI cases) were 3021 (9), 3276 (6), 2364 (4) and the rates were 2.98‰, 1.83‰, and 1.69‰, in 2018, 2019, and 2020 respectively. Notably, the Central-line days (Number of CLABSI cases) in 2021 was 2607 (1) and the CLABSI rate was 0.38‰ in 2021.

### 3.5. Qualitative data

Detailed results for each category are presented below.

#### 3.5.1. Innovation

Twelve interviewees clearly stated that the evidence from the 10 included guidelines was rigorous and strong, and 1 interviewee believed that the evidence needed to be marked with the specific clinical application environment. Fifteen interviewees considered the evidence to be suitable for the ICU. Ten interviewees considered the evidence to be novel. Thirteen interviewees believed that the clinical process does not require major changes. Fourteen interviewees believed that there were no conflicts with interpersonal, psychological, and nursing models. Two interviewees thought that there was no complete synthesis, or that the text translation was too blunt. Five interviewees thought the new processes would enhance patient experience. Two interviewees believed that “evidence support” is a process of continuous social development, and that evidence innovation requires a long period of research.

#### 3.5.2. Affected people

The implementation of the new evidence-based processes was carried out in the ICU. The main populations affected by this were ICU medical staff, newcomers, and medical staff in related departments. Interviewees believed that the new processes will have an impact on newcomers who have not formed a habit, as well as nurses and doctors who have developed some bad habits. Representative statements include:

It will have an impact on medical workers in the ICU and clinical departments involved in contact with central venous catheters. HW #20

It is of great significance to newcomers. HW #21

#### 3.5.3. Views of the evidence

The interviewees’ views of the evidence were very important, and different views on the evidence were apparent. Some interviewees accepted the evidence and thought it was instructive, while some people felt that the evidence was cumbersome. Representative responses included:

This evidence provides a basis for the maintenance and care of clinical central venous catheters and has guiding significance HW #20

Basically, all accept the evidence. HW #18

I feel that it should fit the clinic. How can it be more acceptable to clinical nurses and make them more willing to ask new questions and solve them in time? According to the deep vein maintenance checklist, I feel that the content is too cumbersome. HW #15

#### 3.5.4. Doubt

This summary of evidence was mainly derived from guidelines foreign to China, and shearing, chlorhexidine rubbing, and saline flushing are infrequently used in clinical practice. As such, some doctors, nurses, and researchers expressed certain doubts:

How about flushing the catheter with normal saline every few hours? HW #8

The gauze dressing needs to be replaced in 2 days. The gauze dressings we trained before need to be replaced every 24 hours, or they should be replaced when they are contaminated or leaking. HW #2

Shearing is not specific. HW #21

When replacing the infusion set within 96 hours, consider the airtightness of the system during use. We also have a lot of bolus drugs and frequent disconnections. We did not use a separation membrane; the system is open, so we have to replace the tubing every day. HW #21

Why a single suture? HW #18

What are the advantages of the compound chlorhexidine over other skin disinfectants? Is there a randomized controlled experiment? HW #18

When propofol is infused, the infusion device needs 6 hours to be replaced, which is not clinically possible. HW #18

Wipe for 15 seconds. Few hospitals will do it. HW #18

In terms of patient cleansing, using 2% chlorhexidine to bathe and clean skin every day is unusual, and I don’t know where this recommendation comes from. I need some evidence. It seems that this is not some routine procedure. HW #12

Use stitch-free fixation devices to reduce the risk of infection of intravascular catheters. There are no such things in the deep venous bag. The operability is not so strong in the actual procedure. HW #12

In the summary of recommendations, the first choice is the right internal jugular vein using a ≥10 Fr catheter; placing a non-tunneled CVC recommends the subclavian vein; avoid the tracheostomy. HW #14

#### 3.5.5. Advantages of evidence

The medical staff interviewed clearly believed that the evidence-based recommendations have advantages, and the decreased CLABSI rate confirms that the evidence-based recommendations have advantages.

Compared with the previous treatment method, it is more standardized and more reasonable. HW #8

It can reduce the occurrence of some central venous catheter infections. HW #6

#### 3.5.6. Work efficiency

The interviewees believed that improvements in the following aspects have improved work efficiency.

In fact, you recommend B-ultrasound guidance. We have just started. We should have only started last year. That is, I am in general practice. We are pushing. Let every doctor need it, no matter how familiar you are. The use of ultrasound guidance also improves the success rate of a catheterization, reduces the discomfort of the patient, and reduces the occurrence of complications. HW #13

Puncture under the guidance of color Doppler ultrasound is very useful. It will be more troublesome at first, but it will save time after using it. HW #18

Most of my patients will routinely wipe their bodies with chlorhexidine. HW #18

Checklist is a very good tool to help control quality. HW #14

#### 3.5.7. Recipients

Sixteen interviewees indicated they hope to apply the changes to practice, 12 interviewees think the changes are valuable and meaningful, 7 interviewees clearly pointed out that changes need to be made, 1 said no change is needed, and 7 of the interviewees think the values and beliefs are consistent. The deputy director of the nursing department, the head nurse, and the director of the department supported the development of this research. The department records the daily occurrence of CALBSIs. Eleven interviewees believed that both individuals and teams need training. Four interviewees indicated that individuals have no right to implement changes. Thirteen interviewees believed that individuals are capable of implementing changes. Two key persons interviewed thought that there was basically no resource support.

#### 3.5.8. Conditions for implementing recommendations

The implementation of changes is a clinical process that needs to meet the conditions for clinical change. Interviewees indicated:

Only after learning and training can changes be applied to practice. Our department has a quality management committee, and changes can only be implemented through the quality management committee. HW #14

#### 3.5.9. The meaning of change

Understanding changes is very important, and interviewees expressed the importance of understanding changes.

Change means standardized training, learning relevant theories and practices, and completing the relevant PDCA process. HW #20

It may improve medical behavior, reduce the incidence of deep venous catheter infections, and improve the doctor-patient relationship. HW #13

To be in line with international standards. HW #13

A process of upgrading deep vein treatment, constantly improving, and constantly discovering problems. HW #11

#### 3.5.10. Strategies for change

Change requires a certain strategy, and the clinical medical staff indicated that a clinical process change strategy is necessary.

It is important to strengthen the aseptic concept and the aseptic concept of the daily maintenance of the medical staff! Strengthen manpower, strengthen concepts, and provide strong data evidence support. HW #3

We can start a workshop and let each of us participate in it, instead of just giving lectures. In fact, everyone is more willing to do practical exercises. HW #15

The key people who need support are, of course, the head of the department, and the head nurse. They give orders; everyone’s compliance may be better, right? Our nursing team leader often checks and pays attention to these. How about the compliance of the nurses? Do you follow the procedures strictly? HW #11

#### 3.5.11. Obstacles to change

There are certainly going to be obstacles to change, and the medical staff indicated possible obstacles to change.

Obstacles will definitely be encountered and will increase the workload. But it has just become a habit. HW #8

There are definitely obstacles, mainly compliance. In such a busy medical unit, how can we strictly follow step-by-step, especially in the early stage, according to this recommendation? There are still some difficulties. Mainly, new habits have not been developed yet. HW #13

Some require the support of the equipment department, such as dressing consumables. If they disagree, it is difficult to obtain because the proportion of consumables is now strictly controlled by the hospital. Human resources are very tight, and nurses can’t reach 1:2. HW #14

Different manufacturers of deep venous catheters and accessories, and the quality of different materials are related to the risk of deep venous catheter infection. HW #11

#### 3.5.12. Inner context

Twelve interviewees believed that the nursing department, head nurse, and section director would support the changes. Eight interviewees believed that there is a mechanism to support change in the ICU. A key person interviewed believed that their management was unified and decentralized. Eight interviewees believed that there is a culture of innovation in the ICU. Eight interviewees thought they would actively participate in the change. Four interviewees trusted medical staff to put new ideas into practice. Ten interviewees felt that the plan was valuable after the implementation of the plan. Eight interviewees talked about the experience of ICU reform. Two interviewees believed that this change is consistent with the organization’s strategic focus. Three interviewees felt that there was a lack of organizational support. Two interviewees believed that the ICU has a mechanism to embed changes in daily practice.

In the beginning, the maintenance of the deep vein was not performed with secondary fixation, so adverse events of catheter exposure or fall off occurred. Since the second fixation, the incidence of adverse events in the deep vein, which is the catheter detachment, is significant. Reduced, so there is still experience of change. HW #15

The hospital has very strict control of catheter-related bloodstream infections, and the nursing department is one of the 10 improvement goals of the ICU, so this item is still very important. This change is consistent with the organization’s strategic focus. HW #15

In fact, there are very few opportunities to go out and study. HW #15

Needle-free systems and dressings have been known for 15 years, but now the consumables take up too much space in the cart. HW #14

#### 3.5.13. Outer context

Most of the interviewees were medical staff in the ICU, and they did not have much knowledge about Outer context. A key person interviewed provided some information about Outer context.

#### 3.5.14. Changes are aligned with the strategic priorities of the healthcare system

The country still attaches great importance to the fact that the incidence of catheter infection is a top 3 index and one of the improvement goals of the National Health Commission’s quality control work in 2021. HW #21

#### 3.5.15. Incentives for the healthcare system

There are incentives in the health system to strengthen the proposed changes, and the country has been holding quality improvement competitions at all levels to stimulate change. HW #21

#### 3.5.16. Professional clinical network

China National Database of Nursing Quality (http://cndnq.hqms.org.cn/hospital-admin/nation-data-report/front/index.html). Collecting this indicator (CLABSI infection rate), you can see the national infection rate and the provincial infection rate level. HW #21

## 4. Discussion

We constructed a best evidence-based practice plan for maintenance of CVCs for adult ICU to reduce the incidence of CLABSI.

The evidence used in this study to develop the plan came from guidelines and database literature reviews.^[29]^ Due to the limited timeframe of this study, and the inclusion of 10 guidelines published in 2020,^[23]^ systematic reviews and other types of literature were not included.

In this study, we developed a central venous puncture catheterization process and checklist, and a CVC maintenance process and checklist. The central venous puncture catheterization process can guide the medical staff to perform the procedure as recommended by evidence-based guidelines, and the checklist for the procedure is completed by ICU nurses. It is recommended that when the evidence-based process is implemented, or when a CLABSI occurs after implementation of the evidence-based process that ICU nurses should complete the checklist for 1 to 2 months in order to improve their knowledge and actions. The ultimate goal of the evidence-based practice program that was developed is to improve the clinical outcomes of patients. In this study, the incidence of CLABSI declined markedly after the plan was implemented in 2021. The plan developed included ultrasound-guided catheter placement, chlorhexidine body wash, and the use of a checklist. The adoption of these measures may be an important reason for the rate of CLABSI being reduced. Most interviewees believed that the changes were valuable and meaningful. Some studies have confirmed that the incidence of CALBSI can be maintained at zero for several months or even longer when appropriate measures are implemented.^[6,7,9]^ Further research requires us to maintain the sustainability of our research effects.

Due to time and funding constraints, this evidence-based practice program was implemented in only 1 ICU. The implementation of the program was only for 4 months, and the number of included patients was limited. A long-duration multi-center implementation is necessary to confirm the benefits of the evidence-based practice program that was developed.

## 5. Conclusion

The evidence-based practice program we developed markedly reduced the rate of CLABSI in the ICU, and improved the knowledge and behavior of medical staff of evidence-based recommendations to prevent CLABSI. Interviews were also conducted based on the 4 themes of the i-PARIHS (Innovation, Recipients, Inner context, and Outer context) in order to determine the experiences and views of the medical staff with respect to the program. This project may be used as a reference for similar clinical studies.

## Author contributions

**Conceptualization:** Xiu-wen Chi, Ru He.

**Investigation:** Xiao-heng Wu, Li-juan Wu, Yuan-li Yang, Zhen Huang.

**Writing – original draft:** Xiu-wen Chi.

## Supplementary Material
