# Supplementary material for: Development of best evidence-based practice protocols for central venous catheter placement and maintenance to reduce CLABSI
Source: Medicine (Baltimore). 2024 Jul 5;103(27):e38652. doi: 10.1097/MD.0000000000038652 (PMC11224884; doi:10.1097/MD.0000000000038652)
Supplement: Supplementary file 1 [file medi-103-e38652-s001.docx]

**Table S1. Checklist of central venous catheter placement**

| **Patient Admission Number:** |
| --- |
| **Record time: Year Month Day** |
| **Recorder:** |

| **Content censored** | **Yes, ‘√’**  **No, ‘×’** | **Notes** |
| --- | --- | --- |
| 1. Is the central line in the recommended position?  Yes (cervical, subclavian)  No (femoral vein) |  |  |
| 2. Hand hygiene should be performed before and after catheterization. Use proper hand hygiene, such as hand washing or hand sanitizing. |  |  |
| 3. For indwelling catheters, use maximum aseptic barrier (hat, mask, aseptic gown, aseptic glove, and large aseptic sheet covering the whole body). |  |  |
| 4. When placing the catheter, disinfect the skin with >0.5% chlorhexidine ethanol and let dry. Iodine tincture, iodophor, or 70% ethanol can be used when contraindications to chlorhexidine are present |  |  |
| 5. Placement of the central venous catheter was guided by ultrasound |  |  |
| 6. A single strand suture was used to secure a catheter for short use. Minimize the number of skin suture sites. |  |  |
