# Supplementary material for: Development of best evidence-based practice protocols for central venous catheter placement and maintenance to reduce CLABSI
Source: Medicine (Baltimore). 2024 Jul 5;103(27):e38652. doi: 10.1097/MD.0000000000038652 (PMC11224884; doi:10.1097/MD.0000000000038652)
Supplement: Supplementary file 2 [file medi-103-e38652-s002.docx]

**Table S2. Checklist for central venous catheter maintenance**

| **Patient's Admission Number:** | **Record time:**  **Year Month Day** | **Recorder:** |
| --- | --- | --- |

| Content censored | **Yes, ‘√’**  **No, ‘×’** | **Notes** |
| --- | --- | --- |
| 1. Is the central line placed in the recommended position? Yes (intra-jugular, subclavian), no (femoral vein) |  |  |
| 1. Assess the need for catheter indwelling on a daily basis and immediately remove the catheter when not needed. |  |  |
| 1. Perform hand hygiene before and after catheter maintenance. Use proper hand hygiene, such as hand washing or hand sanitizing. |  |  |
| 1. Observe the site of catheters daily and palpate the dressing. If there is local infection or bloodstream infection, the dressing should be opened for examination. |  |  |
| 1. Is the catheter dressing changed during working shift? (Answer ‘yes’ and fill in the following 4 items truthfully, and do not fill in ‘no’) |  |  |
| 5.1. When dressing replaced, use > 0.5% chlorhexidine ethanol to disinfect the skin and let dry. When there are contraindications, tincture of iodine, iodophor, or 70% ethanol can be selected. |  |  |
| 5.2. The dressing should be replaced at any time when it is wet, lose, or obviously polluted. |  |  |
| 5.3. Gauze dressings |  | Day replaced |
| 5.4. Transparent dressings |  | Day replaced |
| 1. Patients should use 2% chlorhexidine body wash to clean their skin. |  | Chlorhexidine for patients with multi-resistant or pan-resistant infections |
| 1. Flush the tube with sterile saline. |  | □ Flush the tube with heparin saline |
| 1. Seal the tube with heparin saline. |  |  |
| 1. Infusion sets and add-ons for continuous use should be replaced daily. |  |  |
| 1. Use 75% alcohol cotton pads to disinfect connection ends and tubing connections for at least 15 s and allow to dry before use. |  | □ Disinfection time is not enough (15 s) |
